# Doxorubicin catalyses self-assembly of p53 by phase separation

**DOI:** 10.1016/j.crstbi.2024.100133

**Published:** 2024-02-17

**Authors:** Ankush Garg, Gaurav Kumar, Varinder Singh, Sharmistha Sinha

**Affiliations:** a Chemical Biology Unit, Institute of Nano Science and Technology, Sector- 81, Mohali (SAS Nagar), Punjab, 140306, India; b Indian Institute of Science Education and Research, Sector- 81, Mohali (SAS Nagar), Punjab, 140306, India

**Keywords:** p53 protein, Doxorubicin, Phase separation, Self-assembly, Aggregation

## Abstract

Liquid-liquid phase separation plays a crucial role in cellular physiology, as it leads to the formation of membrane-less organelles in response to various internal stimuli, contributing to various cellular functions. However, the influence of exogenous stimuli on this process in the context of disease intervention remains unexplored. In this current investigation, we explore the impact of doxorubicin on the abnormal self-assembly of p53 using a combination of biophysical and imaging techniques. Additionally, we shed light on the potential mechanisms behind chemoresistance in cancer cells carrying mutant p53.

Our findings reveal that doxorubicin co-localizes with both wild-type p53 (WTp53) and its mutant variants. Our *in vitro* experiments indicate that doxorubicin interacts with the N-terminal-deleted form of WTp53 (WTp53ΔNterm), inducing liquid-liquid phase separation, ultimately leading to protein aggregation. Notably, the p53 variants at the R273 position exhibit a propensity for phase separation even in the absence of doxorubicin, highlighting the destabilizing effects of point mutations at this position.

The strong interaction between doxorubicin and p53 variants, along with its localization within the protein condensates, provides a potential explanation for the development of chemotherapy resistance. Collectively, our cellular and *in vitro* studies emphasize the role of exogenous agents in driving phase separation-mediated p53 aggregation.

## Highlights

• Doxorubicin induces aberrant self-assembly of p53 by phase separation.
• Mutations in the p53 induces phase separation of p53.
• Doxorubicin partitioned into p53 mutant condensates which could lead to chemo-resistance.

## Graphical abstract

**Figure:**
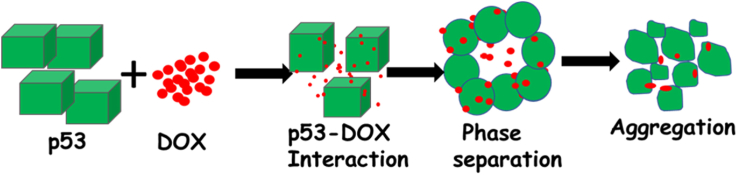
Chemo-therapeutic drug doxorubicin induces aggregation of tumour supressor protein p53.

## 1 Introduction

For many years, protein aggregation has been considered as a hallmark for several neurodegenerative disorders (Soto and Pritzkow, 2018). Recent studies show the presence of aggregates of WTp53 or its mutant forms in several cancer cell lines and tumour biopsy samples indicating a possible link between p53 aggregation and cancer pathogenesis (Ano Bom et al., 2012; De Smet et al., 2017; Freed-Pastor and Prives, 2012; Kanapathipillai, 2018; Levy et al., 2011; Pedrote et al., 2020; Silva et al., 2014; Xu et al., 2011). The effect of the mutations on the p53 aggregation is well documented in several reports and specified as the plausible reason for the loss of tumour suppressive function of the p53 (Ano Bom et al., 2012; Freed-Pastor and Prives, 2012; Pedrote et al., 2020; Silva et al., 2014; Xu et al., 2011). However, the presence of exclusively WTp53 aggregates in cancer cell lines and tumour tissues samples (De Smet et al., 2017; Gong et al., 2015; Yang-Hartwich et al., 2015) raises question if there are factors besides mutation which could contribute towards p53 aggregation.

In recent years, there are several studies which show the effect of the small molecules on the protein aggregation. They have shown that these molecules get accumulated and make a seed which interact with physiological proteins and aggregate them. Some of the examples of the small molecules known for man-made aggregation of proteins are phenylalanine, tyrosine, quinolinic acid, aspartame, drugs etc (Anand et al., 2019; Garg and Sinha, 2022; Sade et al., 2018). Interestingly, the aromatic ring structure is common among all these small molecules. The presence of aromatic rings reminds us of chemotherapeutic drugs which are closely associated with cancer therapeutics and are the first line of treatment for the cancer patients. Existing reports show adverse effects of these chemotherapeutic drugs mainly doxorubicin on physiological proteins such as structural changes and degradation of the proteins (Trynda-Lemiesz, 2004; Zhang et al., 2016). It has also been shown by some studies that administration of doxorubicin increases the level of WTp53. A recent study by transcriptome profiling shows that the administration of doxorubicin results in the accumulation of p53 in the cells which is otherwise degraded at regular interval (McSweeney et al., 2019). In cancer pathogenesis, p53 accumulation, followed by its destabilization and aggregation is observed (Pedrote et al., 2020), however, it is unclear if there is any direct link between p53 aggregation and administration of chemotherapeutic drugs. Based on these intriguing observations, our present study aims to investigate the hypothesis that chemotherapeutic drugs used in cancer treatment could serve as external factors that alter the structure of p53. Additionally, we seek to understand why cancer cells harbouring structural mutant forms of p53 exhibit resistance to chemotherapeutic drugs. To address both concepts, we have chosen doxorubicin as the candidate drug and will utilize wild type p53ΔNterm and its R273 mutants.

## 2 Materials and method*s*

### 2.1 Chemicals

All reagents are of molecular biology grade and procured from Sigma India, unless otherwise mentioned. Ultrapure water is used for all experiments.

### 2.2 Expression and purification of p53ΔNterm variants

The p53ΔNterm variants are expressed and purified as described in (Garg et al., 2020). All the steps of purification are done at 4 °C strictly to ensure purification of native protein and DNase is added at an appropriate step to remove free DNA. The proteins are dialysed in 20 mM Tris Cl, 150 mM NaCl, 2 mM DTT, 5% glycerol buffer before preforming any experiment.

### 2.3 Preparation of Doxorubicin-HCl

Doxorubicin by nature is a hydrophobic anti-cancer drug. For our experiments we have used a water-soluble variant of the drug, Doxorubicin-HCl. The stock of 1 mg/ml of doxorubicin was prepared in milli Q water then subsequently diluted to 0.1 mg/ml (170 μM) in phosphate buffer of pH 7.4 in amber colour vial. This solution is bath sonicate for 15-20 min @ 4 °C and then centrifuge at high speed before further use.

### 2.4 *In vitro* co-localization of p53 and doxorubicin

LN-229 cells, HeLa cells and C-33A cells (obtained from NCCS Pune) are grown on coverslips and treated with 10 μM doxorubicin for 4 h. The cells are fixed with 4% paraformaldehyde and permeabilized by incubating with 0.25% triton-x 100 in PBS for 10 min. After washing, the cells are incubated in blocking buffer (5% BSA in PBS) for 3 h and then incubated overnight with 1:200 dilution of anti-P53 antibody (Bp53-12, Santa Cruz) in blocking buffer at 4 °C. After washing with PBS, cells are incubated with anti-mouse FITC labelled secondary antibody (rat anti-mouse antibody, eBioscience, Invitrogen) for 1 h at room temperature. The nucleus is stained with DAPI. The cells are observed under confocal microscope (LSM 880 model, Carl Zeiss AG, Germany). The colocalization analysis was performed using JACoP (Just another co-localization plugin) and Colocalization Finder Plugin in image j FIJI software. Pearson coefficient was used to measure colocalization of the p53 and the doxorubicin. We have used 4 different images to calculate the Pearson coefficient.

### 2.5 UV-visible spectroscopy

UV-Visible spectroscopy has been used for studying interaction between p53ΔNterm variants and doxorubicin. p53ΔNterm at a concentration of 5 μM was subjected to titration with doxorubicin starting at 1 μM and gradually increasing until reaching a maximum concentration of 20 μM. Similarly, Buffer is titrated with doxorubicin starting from 1uM till 20 μM. Next, we have subtracted the buffer-doxorubicin titration curves from p53-doxorubicin titration curves to get the net change in the p53 absorbance in the presence of doxorubicin. The net change in the absorbance of the p53 in the presence of doxorubicin is shown in results.

### 2.6 Biolayer interferometry

Interactions between the p53ΔNterm variants and doxorubicin at different DOX concentrations (3 μM – 25 μM) are carried out using Forte bio OctateK2 (Molecular Devices, USA). Nickel-NTA biosensors are equilibrated with the protein buffer. This is followed by immobilization of 10 μM His-tagged p53 protein onto the activated sensor. The sensors are then immersed in titer wells containing doxorubicin to record their association kinetics on to the p53ΔNterm variants loaded sensors. The dissociation kinetics are recorded by immersing the sensor tips in p53 dialysis buffer (20 mM Tris-Cl pH 8.0, 150 mM NaCl and 2 mM DTT). The sensors are regenerated using p53 elution buffer containing 50 mM Tris-Cl, 200 mM NaCl and 250 mM imidazole. The association between the protein and drug is estimated based on the changes in the interference pattern of the white light reflected from the reference layer and test layer. Local full fitting of the association and dissociation kinetics is performed using Data Analysis HT 9.0.0.33 software provided with the OctateK2 instrument.

### 2.7 Molecular docking

We have performed p53-DOX docking using AutoDock Vina software. Crystal structure of WTp53 monomer is retrieved from the RCSB (PDB ID: 3TS8) The variants [R273C]p53 and [R273L]p53 are generated *in silico* using pymol and followed by energy minimization. Using AutoDock tools (Trott and Olson, 2009), the structures of WTp53 and its variants were processed during which water molecules were removed from the protein structures and Kollman charges and polar hydrogen atoms were added. The protein structures are then saved as PDBQT files. Then the grid box around the protein structure was generated. The grid size was set at 60, 67, 54 (x, y, z) points with center co-ordinates of 68.849, 12.700, 88.530 (x, y, z) with a grid spacing of 0.619 Å. The grid dimensions were then saved as a text file. Similarly, for the doxorubicin, PDB file of the doxorubicin was opened in the AutoDock tool. Gasteiger charges and polar hydrogen atoms were added to the molecule. All the non-rotatable bonds in the ligand were made flexible using the torsion tree root and it was saved as PDBQT file. To perform the docking, the configuration text file containing the names of the doxorubicin and the p53 file along with the grid dimensions is created. Docking was carried out using Auto Dock Vina program. The binding affinities of the ligand and receptor were obtained in the form of Gibb's free energy. The models with lowest negative Gibbs free energy were further analysed using Ligplot software (Wallace et al., 1995) and the models are generated using Discovery Studio 2020 client.

### 2.8 Dynamic light scattering

The hydrodynamic diameter of the p53ΔNterm variants in the presence and absence of doxorubicin is recorded using Malvern Zeta Sizer. The protein: DOX molar ratio used for DLS study is 1:3 where protein concentration is 5 μM and doxorubicin concentration is 15 μM. The particle size distribution is observed immediately after addition of doxorubicin to the protein sample.

### 2.9 Scattering

To study, the self-assembly of p53ΔNterm variants in the presence and absence of doxorubicin, 5 μM of p53ΔNterm is mixed with 15 μM of doxorubicin (only buffer in control sample) immediately before performing the experiment. The samples are placed in a 1 mm cuvette, and the temperature is maintained at 37 °C (using Peltier) in Edinburg FS5 Spectrofluorometer, (UK). The samples are excited at 500 nm, at a bandwidth of 1 nm and scatter light at 500 nm (bandwidth of 1 nm) is recorded with time.

### 2.10 Circular dichroism

CD measurements are done in a CD spectrophotometer, (Jasco, J-1500, JAPAN). The spectra of 5 μM of p53ΔNterm variants (dissolved in 20 mM Tris-Cl pH 8.0, 150 mM NaCl and 2 mM DTT buffer) in the presence and absence of 15 μM of doxorubicin (are placed in a 0.1 cm path-length quartz cell (Hellma, Forest Hills, NY) is recorded over the wavelength range of 200-260 nm. We cannot go beyond 200 nm due to an increase in the HT above the desired limit of 800 mV, which may affect the PMT. Appropriate blank subtraction has been done for the buffer and the ligand blank spectra prior to analysis.

### 2.11 Atomic Force Microscopy

Samples are imaged in a soft-tapping mode in a Bruker Multimode 8, AFM, Germany with nasoscope controller and j-scanner using silicon carbide tips of 40 N/m force constant. The samples are scanned in an area 2 μm X 2 μm at a scan rate of 0.7 Hz. Five- μM of WTp53ΔNterm proteins are incubated at 37 °C for 2 h in the presence and absence of doxorubicin prior to imaging.

### 2.12 Estimation of Zeta potential

The zeta potential of the p53ΔNterm variants in the presence and absence of doxorubicin is checked using Malvern Zeta Sizer. 5 μM of protein is incubated with different concentrations of doxorubicin for 10 min before recording the zeta potential.

### 2.13 Thioflavin fluorescence

To determine the aggregation of WTp53ΔNterm variants in the presence and absence of doxorubicin, a ThT binding experiment is done. The WTp53ΔNterm protein samples (5 μM) in the presence and absence of doxorubicin (15 μM) is mixed with 20 μM of ThT immediately before performing experiment in Edinburg spectrofluorometer. The samples are excitation at 440 nm and an emission intensity at 490 nm is recorded.

### 2.14 Labelling of proteins

We labelled the protein with NHS-labelled Alexa-488 dye (gives fluorescence in green region). The protein samples are incubated overnight with dye at 4 °C and subsequently buffer exchanged using PD-10 (GE) column to remove free dye. Doxorubicin shows fluorescence in the red region.

### 2.15 Liquid-liquid phase separation

To study liquid-liquid phase separation and liquid droplet formation, the proteins of interest (WTp53ΔNterm and its mutant variants) in the crowded environment i.e. (with 5% PEG-4000) in the presence and absence of doxorubicin are drop-casted on 1% Pluronic's treated glass slides and covered with cover slips. The samples are further visualized under fluorescence microscope (OLYMPUS BX53 microscope) with 20X objective. The proteins are visualized under FITC channel and doxorubicin under Texas Red channel.

## 3 Results

### 3.1 Cellular localization of WTp53 and doxorubicin

**Fig. 1:**
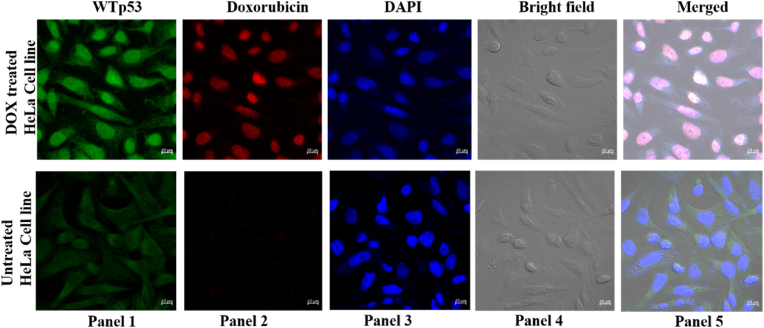
**Co-localization of WTp53 and doxorubicin in cellular nucleus**. Cellular co-localization studies show the increase in the endogenous p53 post doxorubicin treatment where co-localization of WTp53 with doxorubicin is observed in doxorubicin treated HeLa cell lines (a); whereas very low expression of WTp53 is observed in untreated cells where WTp53 localizes in the cytoplasm of the HeLa cell lines (b). All the images are taken at a scale of 10 μm in the confocal microscope where Panel 1 is for WTp53; Panel 2 is for Doxorubicin; Panel3 is for DAPI; Panel 4 is for bright field and Panel 5 is for merged image in a & b.

To validate the previous findings that demonstrated doxorubicin treatment induces p53 over-expression, we have conducted cellular-based experiments using HeLa cancer cells expressing wild-type p53 (WTp53). Initially, the expression of WTp53 in HeLa cells was found to be quite low, as depicted in Fig. 1. However, following treatment with doxorubicin, we have observed a significant increase in WTp53 expression compared to untreated cells, indicating the stimulatory effect of doxorubicin on p53 levels.

Using an Alexa-488 labelled antibody targeting p53 and leveraging the intrinsic red fluorescence of doxorubicin, we have observed that WTp53 co-localizes with doxorubicin within the nucleus of the cells. Quantitative analysis of the colocalization of p53 and doxorubicin was done using JACoP plugin in in image J FIJI software. Pearson coefficient of 0.923 ± 0.015, indicating significant colocalization of the doxorubicin with the p53 protein. The cytofluorogram obtained from the JACoP analysis was shown in supporting figure (Fig. S1). This co-localization prompted us to investigate whether it signifies an interaction between p53 and doxorubicin.

To address this question, we have employed biophysical techniques to assess the interaction between p53 and doxorubicin. Notably, we have conducted these experiments with a truncated form of p53, p53ΔNterm (residues 82-393), as it exhibited better yield and stability, as previously reported (Garg et al., 2020). This choice allowed us to perform robust biophysical investigations and gain insights into the interaction between p53 and doxorubicin without the complication of protein instability observed in full-length p53.

### 3.2 Interaction between WTp53ΔNterm and doxorubicin

**Fig. 2:**
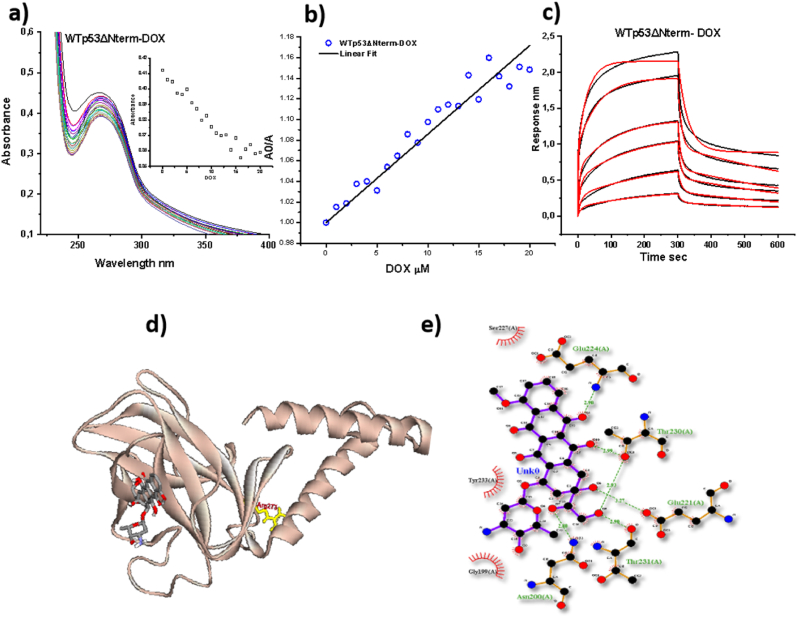
**Interaction between p53 and doxorubicin**: UV-visible spectra of WTp53 showing net decrease in the absorbance with increasing concentration of doxorubicin (a), Inset shows change in the absorbance of p53 at 280 nm with increasing doxorubicin concentration (inset, a); Linear plot of *A*_*0*_*/A = X. B + C*, where *A*_*0*_ is the absorbance of p53 without doxorubicin and A is the absorbance of the p53 with increasing concentration of doxorubicin and X represents doxorubicin concentration (b); BLI results showing the association and dissociation kinetics for the interaction of DOX with WTp53ΔNterm where black is the original curve and red is the fitted data (c); Docking of p53 with the doxorubicin showing best fit docked models of WTp53-DOX with binding affinity of WTp53-DOX is -7.2 kcal/mol (d); The residual level interactions of DOX with WTp53 identified using Ligplot – server (e). (For interpretation of the references to colour in this figure legend, the reader is referred to the Web version of this article.)

We have checked the interaction between doxorubicin and WTp53ΔNterm by probing the change in the absorption spectra of the WTp53ΔNterm in the presence of doxorubicin. We have observed net decrease in the absorbance at 280 nm post doxorubicin addition (Fig. 2a). Next, we have plotted the ratio of the absorbance of WTp53ΔNterm in the absence of doxorubicin (A_0_) to the absorbance of WTp53ΔNterm in the presence of different concentrations of doxorubicin (A) v/s concentration of doxorubicin (Fig. 2b). We have observed a curve which fitted best with the linear equation *A0/A = X. B + C*, where X = DOX concentration and B is the slope which gives binding constant and C is the intercept whose value is 1. We have determined the dissociation constant K_D_ i.e., inverse of the binding constant as 11.64 x10^-5^ M from the fitted data indicating weak binding between doxorubicin and WTp53ΔNterm. Further we have confirmed the interaction between WTp53ΔNterm and doxorubicin using biolayer interferometry technique (BLI) (Fig. 2c). Here, we have used the 6x histidine tag of the WTp53ΔNterm to immobilize the protein on to nickel-NTA sensor and then titrated it with different concentrations of doxorubicin i.e., from 3 μM – 25 μM for test sample and used buffer instead of doxorubicin as a control reaction and observed the association and dissociation kinetics. An increased in the doxorubicin concentration led to an enhanced association response. The association-dissociation curves obtained fitted best with 1:2 heterogeneous model which suggested the presence of heterogeneous population of proteins. This might be attributed to self-assembly nature of these proteins. The average dissociation constant obtained from all the local fitting of the curves is *K*_d1=_ 3.4 x10^-5^ ± 0.1x 10^-5^ M and *K*_d2_ = 2.4x10^-6^ M ± 0.1x 10^-6^ M respectively. This indicated that one of the populations showed weak binding with doxorubicin whereas other showed comparatively moderate to strong affinity. To understand the residues involved in the interaction between WTp53ΔNterm and doxorubicin, we have carried out molecular docking of WTp53 monomer (N-terminal is unstructured and hence cannot be crystallized) (PDB ID: 3TS8) with doxorubicin using Auto Dock Vina software (Trott and Olson, 2009). We have obtained probable multiple binding models of WTp53-doxorubicin with different binding energies from the docking results. The best model with lowest ΔG value was chosen for further residual level analysis as shown in Fig. 2d. The residues of WTp53 interacted with doxorubicin were identified using Ligplus software (Wallace et al., 1995) and we have observed a combination of hydrophobic and hydrogen bond interactions between doxorubicin and WTp53. Doxorubicin showed hydrophobic contact with Gly-199; Ser-227; Tyr-233 residues and it participated in hydrogen bonding with Asn-200; Glu-221; Glu-224; Thr-230 and Thr-231 residues with a binding affinity of -7.2 kcal/mol (Fig. 2e &Table S1). In the next section, we have investigated the consequences of these interactions on the structure of WTp53.

### 3.3 Doxorubicin induces associate formation in WTp53ΔNterm

**Fig. 3:**
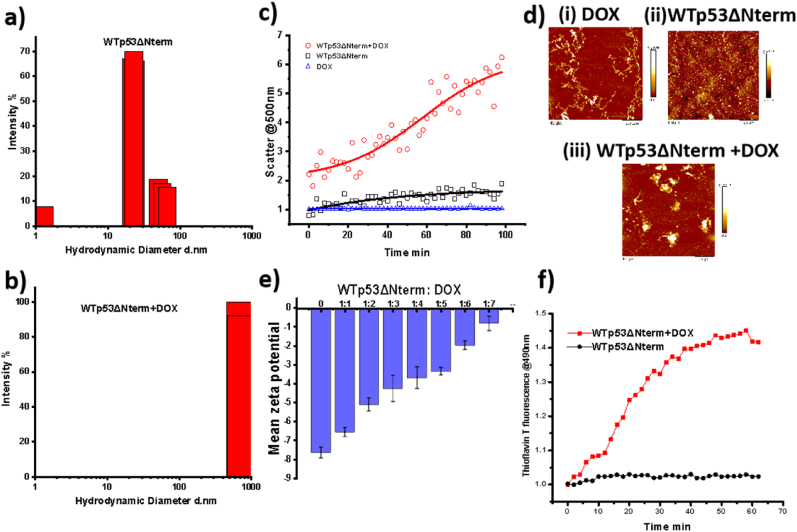
**Doxorubicin induces self-assembly of p53:** Hydrodynamic diameter of WTp53ΔNterm in the absence (a) and in the presence of doxorubicin at 25 °C (b); Time course scattering intensity@ 500 nm of WTp53ΔNterm in the absence (black) and in the presence (red) of doxorubicin @ 37 °C, where doxorubicin is represented as blue curve (c); Atomic force microscopy micrographs of the DOX alone (d(i)), WTp53ΔNterm alone (d(ii)) and WTp53ΔNterm -DOX complex (d(iii). Scale used in each figure (d) is 2 μm; The change in the mean zeta potential of the WTp53 with increasing concentration of doxorubicin (e); Time course change in the thioflavin T fluorescence of WTp53ΔNterm in the presence (red curve) and absence of doxorubicin (black curve) (f). (For interpretation of the references to colour in this figure legend, the reader is referred to the Web version of this article.)

To understand the effect of the doxorubicin on WTp53ΔNterm assembly, we have used 1:3 ratio of protein: doxorubicin where WTp53ΔNterm concentration is 5 μM and doxorubicin concentration of 15 μM. We have checked the hydrodynamic diameter of WTp53ΔNterm in the absence (Fig. 3a) and presence of doxorubicin (Fig. 3b) using dynamic light scattering experiment. The immediate increase in the hydrodynamic diameter of the WTp53ΔNterm from nanometres to micrometres post addition of doxorubicin suggested increased assembly of p53. These quaternary assemblies were not associated with any significant secondary structural changes as shown by circular dichroism studies (Fig. S2a). Next, we have seen time dependent change in the WTp53ΔNterm association in the presence and absence of doxorubicin at physiological temperature using scatter experiment (Fig. 3c). We have not observed any significant increase in the scatter intensity of WTp53ΔNterm (**black curve in** Fig. 3c). However, presence of doxorubicin has increased the scatter intensity of WTp53 by almost 6 folds indicating doxorubicin induced associate formation of the WTp53ΔNterm (red curve in Fig. 3c). Interestingly, the zero-point scatter intensity of WTp53ΔNterm in the presence doxorubicin was also higher than only WTp53ΔNterm indicated immediate effect of doxorubicin on WTp53ΔNterm associate formation. The protein samples at the end of the scatter experiment were subjected to circular dichroism (Fig. S3a). Significant secondary structural changes in the protein samples were observed indicating doxorubicin induces WTp53ΔNterm destabilization with time. To understand the morphology of the assemblies formed, the samples at the end of the scatter experiment were subjected to atomic force microscopy (AFM). We have observed fibrillar morphology of the doxorubicin (Fig. 3d (i)) whereas very small nanometre range globular assemblies were observed for WTp53ΔNterm (Fig. 3d (ii)). The WTp53ΔNterm converted to micron ranged assemblies in the presence of doxorubicin where we observed bigger assemblies in complexation with the fibrillar assemblies which suggests that doxorubicin interact with WTp53ΔNterm leading to the formation of higher order WTp53ΔNterm-dox complexes (Fig. 3d (iii)). The decrease in the zeta potential of the WTp53ΔNterm from negative charge to zero with increasing concentration of doxorubicin from 5 μM to 35 μM further supported that doxorubicin destabilized the structure and induces aggregation of WTp53ΔNterm (Fig. 3e). Earlier reports indicated that p53 and its mutant variants form ThT-positive aggregates (Ano Bom et al., 2012), to test if these associates developed a typical amyloidogenic cross-β structure, we have next performed a time course aggregation assay of the WTp53ΔNterm variants probed by ThT. The presence of doxorubicin increased the amyloidogenicity as indicated by time dependent increase in the thioflavin T fluorescence of WTp53ΔNterm in the presence of doxorubicin compared to WTp53ΔNterm control (Fig. 3f). Altogether, amalgamation of spectroscopic and imaging techniques suggested that doxorubicin triggered the aberrant self-assembly of WTp53ΔNterm.

### 3.4 Co-phase separation of the p53-doxorubicin lead to aggregation

**Fig. 4:**
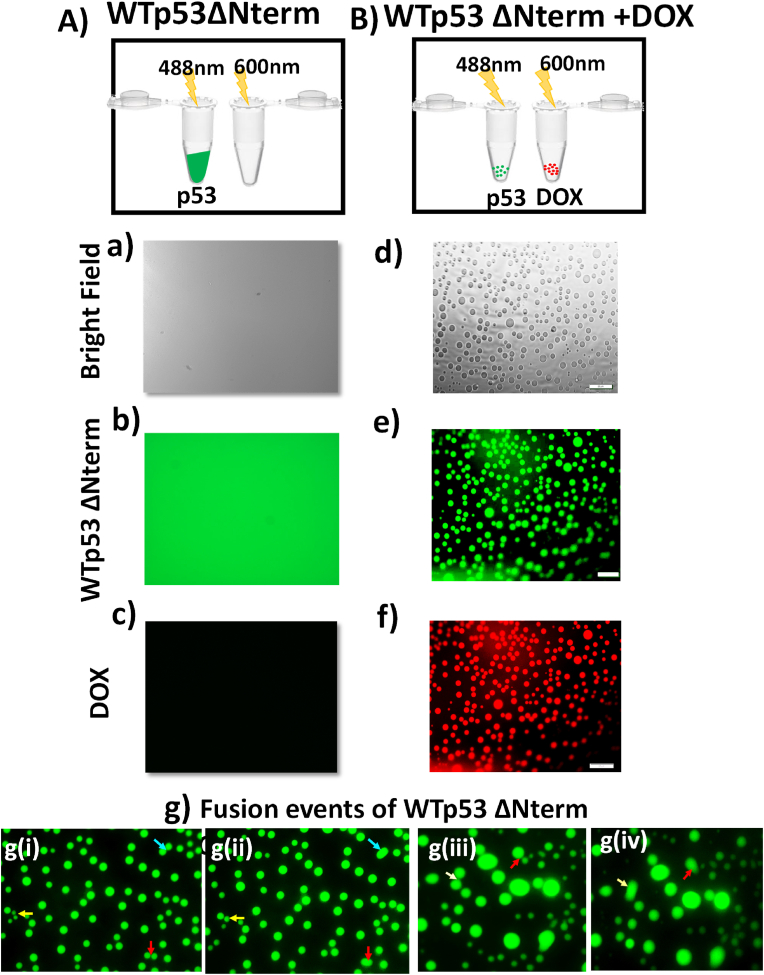
**Phase separation of WTp53 in the presence of doxorubicin**. Schematic showing the experimental procedure for the fluorescence microscopy of p53 in the absence (A) and presence (B) of doxorubicin in crowded environment. WTp53ΔNterm (labelled with Alexa-488 dye) + PEG in the bright Field (a), in FITC channel (b), and in Texas red channel (no fluorescence) (c); WTp53ΔNterm + PEG + DOX in the bright field channel (d); in the FITC channel (e), and in the Texas Red channel (f). The fusion events of the droplets of WTp53ΔNterm + PEG + DOX (g) where one of the fusion sets is shown in g(i-ii) and another set is shown in g (iii-iv). Scale used is 20 μm. (For interpretation of the references to colour in this figure legend, the reader is referred to the Web version of this article.)

To understand the dynamics going on into the solution, we have performed fluorescence microscopic studies where we have labelled the WTp53ΔNterm with alexa-488 dye and have used the intrinsic fluorescence of doxorubicin in the red region to probe the interaction between WTp53ΔNterm and doxorubicin. We have not observed any assemblies of WTp53ΔNterm at a concentration range of 5 μM-15 μM (data shown at a concentration of 15 μM) at 25 °C. To induce phase separation, as a standard practice PEG molecule was used. Here, we have used 5% of PEG-4000 molecular weight for all the microscopic studies. The presence of PEG-4000 mimicked the intracellular environment by maintaining the viscosity equivalent to cell which is provided by high concentration of macromolecules in the cell (Boyko et al., 2019; Julius et al., 2019; Kanaan et al., 2020; Lin et al., 2015; Park et al., 2020; Singh et al., 2020). Interestingly, WTp53ΔNterm has not shown any phase separation in the presence of 5% PEG-4000 (Fig. 4(A, (a-c)) (Kamagata et al., 2020). Further, we have investigated the effect of doxorubicin on the phase transition of WTp53ΔNterm. We have observed that WTp53ΔNterm +5% PEG-4000 formed spherical associates in the presence of doxorubicin which gives green fluorescence corresponding to Alexa-488 labelled WTp53ΔNterm and red intrinsic fluorescence for doxorubicin (Fig. 4d-f). These spherical associates were actually WTp53ΔNterm protein droplets indicated from the time dependent fusion of these spherical entities (Fig. 4g) (Kanaan et al., 2020). This suggested that doxorubicin induced liquid-liquid phase separation of WTp53ΔNterm leading to the formation of liquid droplets. In supporting section, we have elaborated it by using different ratio of WTp53ΔNterm protein: DOX from 1:1 to 1:4 molar ratio to understand the effect of the doxorubicin on the WTp53ΔNterm assembly. At a molar ratio of 1:1 of protein and doxorubicin, we have observed very few droplets which gave fluorescence in both the green region and red region but most of the protein was in solution. The increase in the number of the droplets with an increasing ratio of DOX: WTp53ΔNterm was observed as shown in Fig. S4. At these concentrations of doxorubicin used above, doxorubicin has not shown any self-assembly as indicated by turbidity experiments (Fig. S5) as well by microscopy experiment (Fig. S6). Altogether, our results showed that doxorubicin interacted with WTp53ΔNterm and induced aggregation which is mediated by a phase transition event.

### 3.5 Correlation between p53 mutations and drug resistance

It has been well documented that presence of mutant form of p53 in cancer cell line induced resistance of the cancer cell lines towards chemotherapeutic drugs (Hientz et al., 2017; Huang et al., 2019; Zhou et al., 2019). This chemo-resistance effect was shown by both the DNA-contact and structural mutant of p53. The increase in the transcription of the several genes responsible for multiple drug resistance was one of the well-known mechanisms for the chemo-resistance by the DNA-contact mutants (Chan and Lung, 2004; Preudhomme et al., 1993). However, the role of structural destabilized mutants in the chemo-resistance is still not clear. To gain insight into the effect of the structural destabilization on chemoresistance in cancer, we have chosen [R273C]p53 and [R273L]p53 mutants, which are very frequently found in cancer.

#### 3.5.1 Interaction between p53 mutants and doxorubicin

**Fig. 5:**
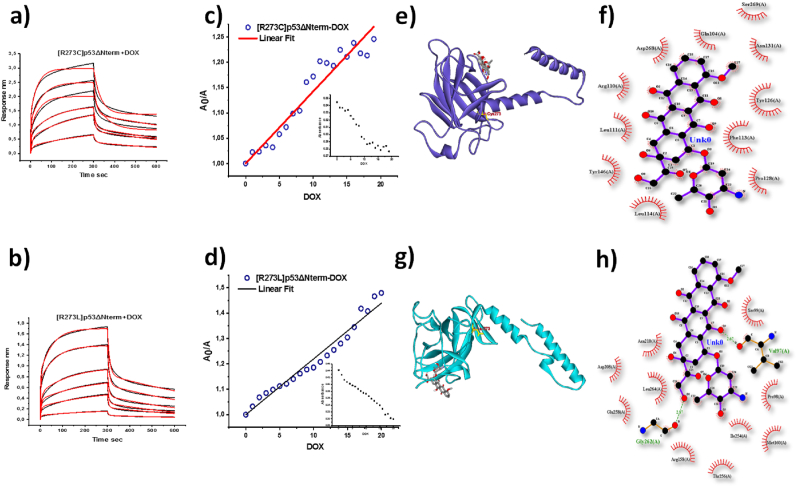
**Interaction of the p53 mutants and doxorubicin:** BLI results showing the association and dissociation kinetics for the interaction of DOX with [R273C]p53ΔNterm (a) and with [R273L]p53ΔNterm (b) where black is the original curve and red is the fitted data; Linear plot of the Linear plot of *A*_*0*_*/A = X. B + C*, where *A*_*0*_ is the absorbance of [R273C]p53ΔNterm without doxorubicin and A is the absorbance of the [R273C]p53ΔNterm with increasing concentration of doxorubicin and X represents doxorubicin concentration (c), where inset shows decrease in the absorbance of [R273L]p53ΔNterm at 280 nm with increasing concentration of doxorubicin; Linear plot of the Linear plot of *A*_*0*_*/A = X. B + C*, where *A*_*0*_ is the absorbance of [R273L]p53ΔNterm without doxorubicin and A is the absorbance of the [R273L]p53ΔNterm with increasing concentration of doxorubicin and X represents doxorubicin concentration (d), where inset curve shows decrease in the absorbance of [R273L]p53ΔNterm at 280 nm with increasing concentration of doxorubicin; Docked model of the [R273C]p53ΔNterm and doxorubicin (e); along with the residual interactions between [R273C]p53ΔNterm and doxorubicin represented in (f); Docked model of the [R273L]p53ΔNterm and doxorubicin (g) along with the residual interactions between [R273L]p53ΔNterm and doxorubicin represented in (h). (For interpretation of the references to colour in this figure legend, the reader is referred to the Web version of this article.)

We have aimed to investigate the influence of these mutant p53 variants on chemotherapy, with a focus on their interaction with doxorubicin as a potential therapeutic agent. We have checked the interaction of these variants with doxorubicin using BLI. BLI study showed that [R273C]p53ΔNterm (Fig. 5a) and [R273L]p53ΔNterm (Fig. 5b) interacted with doxorubicin where the interaction of [R273C]p53ΔNterm with doxorubicin is comparable with [R273L]p53ΔNterm. We observed 2:1 heterogeneous binding indicating the presence of more than one population in the solution. The dissociation constants for [R273C]p53-doxorubicin obtained from the fitted curves are *K*_d1=_ 2.2 x10^-6^ M ± 0.05 x10^-6^; *K*_d2_ = 4.2x10^-6^ ± 0.05x10^-6^ whereas the dissociation constant for [R273L]p53ΔNterm -doxorubicin is *K*_d1=_ 6.4 x10^-6^ ± 0.7x 10^-6^ M; *K*_d2_ = 4.4 x10^-6^ ± 0.5x 10^-6^M. Both the dissociation constants in the mutant variants were less than 10 μM range as compared to WTp53ΔNterm where we have observed one with less than 10 μM and other in the range of 30 μM indicating stronger interaction of the mutants with the doxorubicin compared to WTp53ΔNterm. Similarly, we have also probed the interaction by observing the change in the absorbance at 280 nm with titration of doxorubicin. We have observed net decrease in the absorbance of [R273C]p53ΔNterm (Fig. 5c inset) and [R273L]p53ΔNterm (Fig. 5d inset) in the presence of doxorubicin. Next, the ratio of the absorbance of mutant p53 in the absence of doxorubicin (A_0_) to the absorbance of p53 in the presence of different concentrations of doxorubicin (A) is plotted v/s concentration of doxorubicin as shown in Fig. 5c & d for [R273C]p53ΔNterm and [R273L]p53ΔNterm respectively. The dissociation constants obtained from the 1/slope of the fitted curves was 70 x 10^-6^ M for [R273C]p53ΔNterm and 45 x 10^-6^ M for [R273L]p53ΔNterm which also indicated stronger interaction of the mutants compared to WTp53ΔNterm. Further, we have looked for the residues responsible for interaction of mutants with doxorubicin using Auto Dock Vina software. Most of the residues of [R273C]p53 and [R273L]p53 participated in the hydrophobic interaction with doxorubicin. Gln-104; Arg-110; Leu-111; Phe-113; Leu-114; Tyr-126; Pro-128; ASN-131; Tyr-146; ASP-268; SER-269 were the residues of [R273C]p53 that made hydrophobic contact with doxorubicin (Fig. 5e & f) (Table S1). Pro-98; Ser-99; Met-160; Asp-208; Asn-210; Arg-158; Ile-254; Thr-256; Glu-258; Leu-264 were the residues of [R273L]p53 that involved in the hydrophobic interaction with doxorubicin whereas very few residues i.e., Val-97 and Gly-262 of [R273L]p53 took part in the hydrogen bonding with doxorubicin (Fig. 5g & h) (Table S1). The difference in the type of interaction and the interaction sites among variants could be due to the structural perturbations that occurred due to substitution at R273 position. This is in support to the our previous observation where we showed the substitution of R273 position altered the structural properties (Garg et al., 2020). The residues of [R273C]p53 and [R273L]p53 which were responsible for doxorubicin interaction are present in the vicinity of the aggregation prone region of p53. This implied that the exposure of the certain aggregation prone residues from 250 to 260 which were comparatively buried in WTp53 (Ghosh et al., 2014). We confirmed this by computing the solvent accessible surface area (SASA) of the aggregation prone region using pymol software (Schrödinger, 2010). The SASA of PILTIITL region in WTp53ΔNterm is 44 Å^2^ which significantly increased to 85 Å^2^ and 96 Å^2^ for [R273C]p53 ΔNterm and [R273L]p53 ΔNterm respectively (Schrödinger, 2010)

#### 3.5.2 Doxorubicin further accelerates the aggregation of p53 mutants

**Fig. 6:**
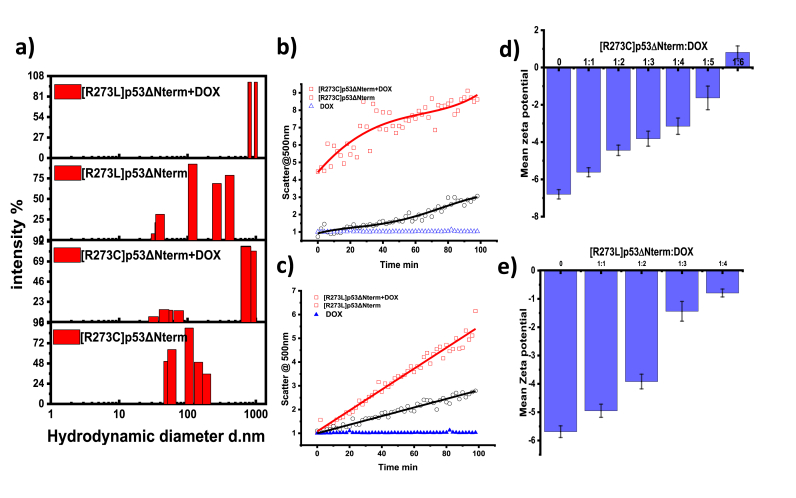
**Doxorubicin accelerates the association of p53 mutants.** Hydrodynamic diameter of [R273C]p53ΔNterm and [R273L]p53ΔNterm in the absence and presence of doxorubicin (a); Time course scatter of the [R273C]p53ΔNterm in the absence (black) and in the presence of doxorubicin (red) (b); Time course scatter of the [R273L]p53ΔNterm in the absence (black) and in the presence of doxorubicin (red) (c); Change in the zeta potential of the [R273C]p53ΔNterm with increasing concentration of doxorubicin (d); Change in the zeta potential of [R273L]p53ΔNterm with increasing concentration of doxorubicin (e). (For interpretation of the references to colour in this figure legend, the reader is referred to the Web version of this article.)

Subsequently, we explored how the interaction influenced the assembly formation of mutant proteins. We assessed the hydrodynamic size of the R273ΔNterm variants and noted a hydrodynamic size falling within the 300-500 nm range, indicating the mutant's propensity for self-assembly (Fig. 6a). This was in accordance with our previous study where we have shown that [R273C]p53ΔNterm and [R273L]p53ΔNterm induce structural changes and can be classified as structural mutants (Garg et al., 2020). The addition of doxorubicin further increased the hydrodynamic diameter to micron range. This implied that doxorubicin interacted with the mutant variants, prompting additional association. Subsequent circular dichroism studies indicated that the secondary structure of mutant [R273C]p53ΔNterm and [R273L]p53ΔNterm in the presence and absence of doxorubicin remained comparable at sub-optimal temperatures (Fig. S2b & S2 c). This implies that higher order assemblies of p53 formed immediately post addition of doxorubicin without significant structural perturbation. K2D2 analysis further explained the differences in the percentage of helical and beta sheet for the WTp53 ΔNterm and its mutant variants [R273C]p53ΔNterm and [R273L]p53ΔNterm (Fig. S2). Next, we performed the time dependent scatter experiment to monitor the time course association. The initial scatter intensity of [R273C]p53 ΔNterm and [R273L]p53 ΔNterm was higher as compared to WTp53ΔNterm which indicated that the mutants have higher tendency to self-assemble. The presence of doxorubicin induced higher order assemblies in all the variants of p53 as indicated from increased time dependent scatter intensity (Fig. 6b & c). The mutants at the end of the scatter experiment were subjected to circular dichroism which indicated destabilized secondary structure both in the presence and absence of doxorubicin (Figs. S3b and S3c). Further zeta potential studies showed the overall decrease in the negative surface charge of the protein in the presence of doxorubicin indicating the associate formation (Fig. 6d & e). It has been observed that a molar ratio of 1:5 for [R273C]p53ΔNterm protein: DOX and 1:4 for [R273L]p53ΔNterm: DOX compared to 1:7 ratio for WTp53ΔNterm: DOX was required to decrease the zeta potential to zero.

#### 3.5.3 Mutation in p53 also induces phase separation

It is well documented in the literature that mutations in the p53 destabilize the structure and induce aggregation. Recently, several groups have shown that the aggregation of the p53 is mediated by the phase separation event where these destabilizing mutants undergo liquid-liquid phase separation which eventually formed irreversible aggregates (Petronilho et al., 2021; Silva et al., 2023; Yang et al., 2021). In our previous work, we have shown that previously mentioned well known DNA-contact mutations i.e., [R273C]p5ΔNterm and [R273L]p53ΔNterm destabilized the p53 structure by different mechanism and hence categorized as structured mutants (Garg et al., 2020). In the present work, we have observed that Alexa-488 labelled [R273C]p53ΔNterm in the presence of 5% PEG-4000 undergoes liquid-liquid phase separation as shown by the presence of spherical droplets (Fig. S7a) where we observed green fluorescence corresponded to [R273C]p53ΔNterm. The liquid nature of the phase separated [R273C]p53ΔNterm was further confirmed by fusion of these spherical droplets (Fig. S7b). [R273L]p53ΔNterm formed solid assemblies in the crowded environment as seen under bright field and fluorescence channel (Fig. S8a). This transition in the phase separation from liquid droplets in [R273C]p53ΔNterm to solid assemblies in [R273L]p53ΔNterm respectively could be due to higher aggregating tendency of [R273L]p53ΔNterm compared to [R273C]p53ΔNterm. Further, we have observed that [R273L]p53ΔNterm formed liquid-liquid droplets at very low temperature (18 °C) (Fig. S8b) which showed transition to solid assemblies at room temperature. These phase separating entities evolved to irreversible amyloidogenic associates at 37 °C i.e., physiological temperature as reported in previous study (Garg et al., 2020). This indicated that mutations in p53 induced phase separation which eventually leads to aggregation.

#### 3.5.4 Partitioning of doxorubicin into mutant p53 condensates

**Fig. 7:**
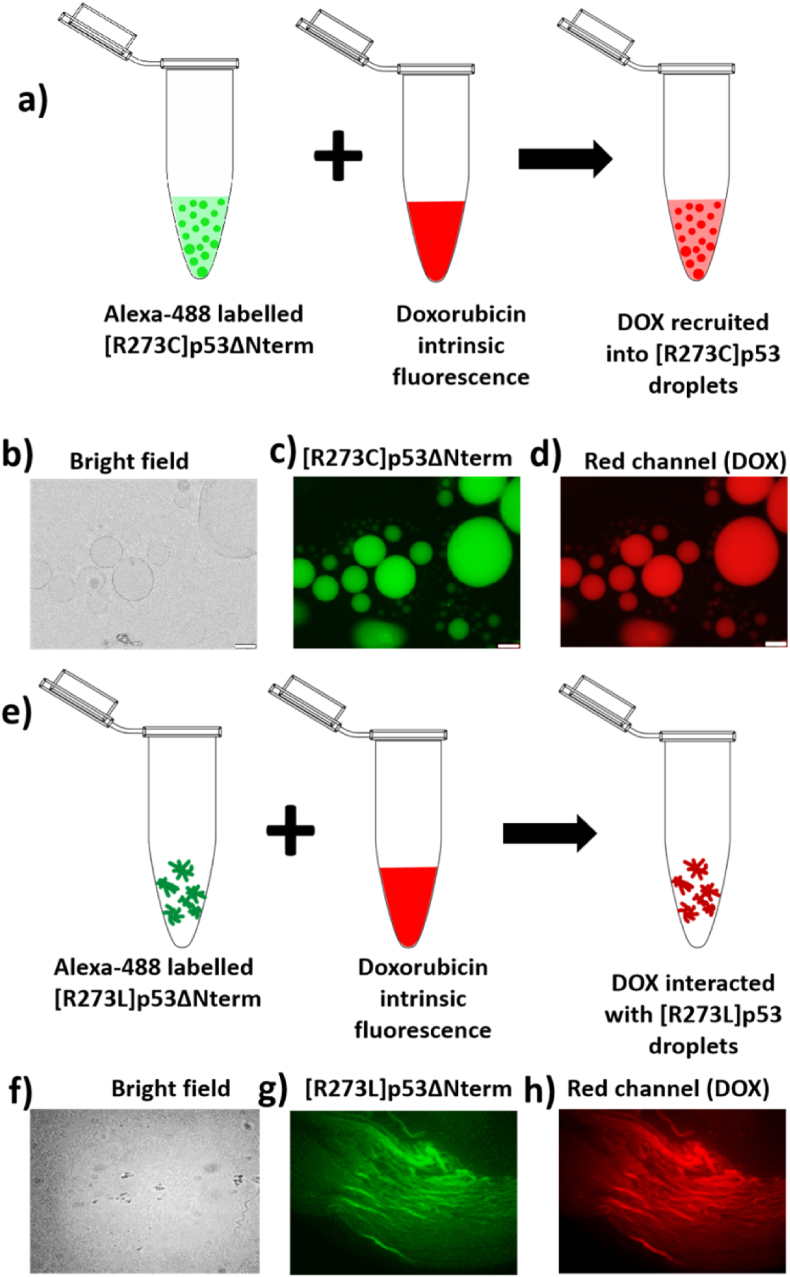
**Sequestration of doxorubicin within phase separated R273 variants**. Schematic representation of partitioning of doxorubicin into pre-formed [R273C]p53ΔNterm condensates (a); [R273C]p53ΔNterm + PEG-4000+DOX in the bright field (b), [R273C]p53ΔNterm in the FITC channel (c) and DOX in the TEXAS Red channel (d); Schematic representation of interaction of doxorubicin with [R273L]p53ΔNterm associates (e); [R273L]p53ΔNterm + PEG + DOX in the bright field (f), [R273L]p53ΔNterm in the FITC channel (g) and DOX in the TEXAS red channel (h). Scale used is 20 μm. (For interpretation of the references to colour in this figure legend, the reader is referred to the Web version of this article.)

Next, we have seen that what happens if we added doxorubicin into the solution containing phase separated [R273C]p53ΔNterm as shown by schematic Fig. (7a). In presence of doxorubicin, we have seen both the red and green fluorescence in the droplets suggesting the recruitment of the doxorubicin in the mutant [R273C]p53ΔNterm protein droplets (Fig. 7b-d). This indicated that doxorubicin interacted with the mutants and got recruited into the condensates. Further, we have observed that with the addition of doxorubicin to the liquid-solid assemblies of [R273L]p53ΔNterm, we started observing fluorescence for both doxorubicin and alexa-488 label protein indicating interaction between the assemblies and doxorubicin (Fig. 7e (schematic), 7(f-h).

In the next set of experiments, we have performed cell culture studies using C-33A cancer cell line harbouring the R273C mutation. We have observed more expression of [R273C]p53 in the cancer cell line compared to WTp53 suggesting overexpression of mutant form of p53 in the cancer cell line. We have detected some puncta formation in the cancer cell line indicating over-expression and aggregation of mutant form of p53 in the cancer cell line (Fig. S9a). Upon, treatment of doxorubicin, we have observed doxorubicin gets co-localized in the puncta of [R273C]p53 (Fig. S9b). Altogether our biophysical and fluorescence microscopy studies suggested that doxorubicin interacted with the p53 mutants and get partitioned into their condensates. This could be one of the plausible mechanisms opted by structural mutant to make the cancer cells resistant towards chemotherapeutic drugs.

## 4 Discussion

Of late increasing body of literature suggest that liquid-liquid phase separation is crucial for several key physiological processes. The liquid-liquid phases developed due to the alterations in the internal environment of the cell leads to the formation of membranelles organelles that shape up the fate of several cellular processes (Alberti and Dormann, 2019; Alshareedah et al., 2019; Kanaan et al., 2020; Shakya et al., 2020; Singh et al., 2020). Nonetheless, the role of the any external stimuli associated with these physiological events are not explored meticulously. In the present manuscript, we provided experimental evidence in support of the notion that there exists a direct link between p53 aggregation and chemotherapeutics. We have shown that doxorubicin, a commonly used chemotherapeutic drug interacts with WTp53ΔNterm, where our molecular docking studies suggested that these interactions are mediated through both ionic interactions and hydrophobic contact and promotes the aggregation of protein. Earlier we have shown that [R273C]p53ΔNterm and [R273L]p53ΔNterm are structural mutants which destabilized the structure and triggered the aggregation of p53. The tendency of [R273C]p53ΔNterm to undergo liquid-liquid phase separation and liquid-solid transition in [R273L]p53ΔNterm indicated that phase separation is the principal mechanism or driving force behind their aggregation. This also suggested that variants generated from the same codon also have different self-assembly properties which is in accordance with our previous report (Garg et al., 2020).

We have made an observation that doxorubicin gets sequestered in the liquid-like droplets of [R273C]p53ΔNterm. Our observations are in support with the recent report by Klein et al., (2020), where the authors have shown the partitioning of the cancer chemotherapeutic drugs within the nuclear condensates (Klein et al., 2020). The strong interaction and the sequestration of the doxorubicin in the p53 mutant droplets of might be one of the explanations for the chemo-resistance gain-of function shown by the p53 mutants as the incorporation of doxorubicin in the protein droplets actually decrease their local concentration at the target site (Brachova et al., 2013; Freed-Pastor and Prives, 2012; Hientz et al., 2017; Huang et al., 2019). This idea is in support with recent report by Genovese et al., showed the strong binding of doxorubicin to sorcin protein and that confered chemo-resistance of the cancer cells (Genovese et al., 2017).

Together our study established a new link between p53 aggregation and cancer where we found that cancer treatment may also affect the protein stability and promote self-assembly. These drugs act as double-edged sword where at one side they prevent the proliferation of cancerous cells, on the other hand, they adversely affect the stability of p53 protein, which plays a pivotal role in the cancer biology. Appropriate therapeutic strategies need to be developed that can address such side effects of the widely used chemotherapeutic drugs.

## Funding

Institutional research grant.

## CRediT authorship contribution statement

**Ankush Garg:** Data curation, molecular biology, cell culture, spectroscopy, Formal analysis, Writing – original draft. **Gaurav Kumar:** Data curation, spectroscopy. **Varinder Singh:** Data curation, cell culture experiments. **Sharmistha Sinha:** Conceptualization, Methodology, Software, Supervision, Investigation, Writing – review & editing.

## Declaration of competing interest

The authors declare that they have no known competing financial interests or personal relationships that could have appeared to influence the work reported in this paper.

## Data Availability

Data will be made available on request.
