# Supplementary material for: Doxorubicin catalyses self-assembly of p53 by phase separation
Source: Curr Res Struct Biol. 2024 Feb 17;7:100133. doi: 10.1016/j.crstbi.2024.100133 (PMC10906149; doi:10.1016/j.crstbi.2024.100133)
Supplement: Multimedia component 1 [file mmc1.docx]

**Supporting information**

**Doxorubicin catalyses self-assembly of p53 by phase separation**

*Ankush Garg^1^, Gaurav Kumar^1^, Varinder Singh^2^, Sharmistha Sinha^1^**

^1^Chemical Biology Unit, Institute of Nano Science and Technology, Sector- 81, Mohali (SAS Nagar), Punjab, India, 140306

^2^ Indian Institute of Science Education and Research, Sector- 81, Mohali (SAS Nagar), Punjab, India, 140306

**[*sinhas@inst.ac.in](mailto:*sinhas@inst.ac.in)**

**Supporting Figures**

**a)**

**b)**

**c)**

**
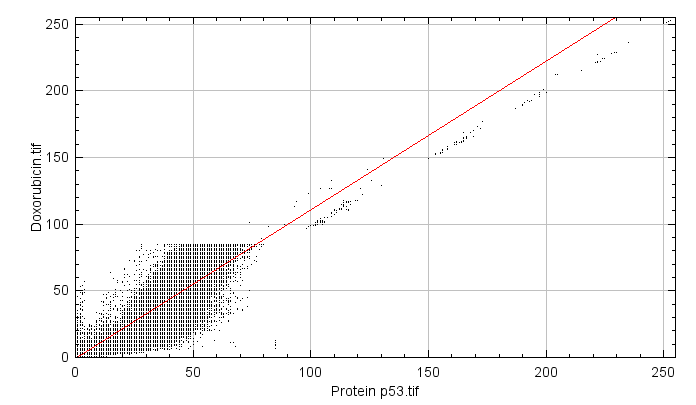
**

**Figure S1**: Cytofluorogram between p53 protein and doxorubicin. Quantitative analysis of co-localization of p53 and doxorubicin was done by image J analysis using JACoP plugin. This analysis indicated positive co-localization between p53 and doxorubicin with pearson coefficient of 0.923 ±0.015.


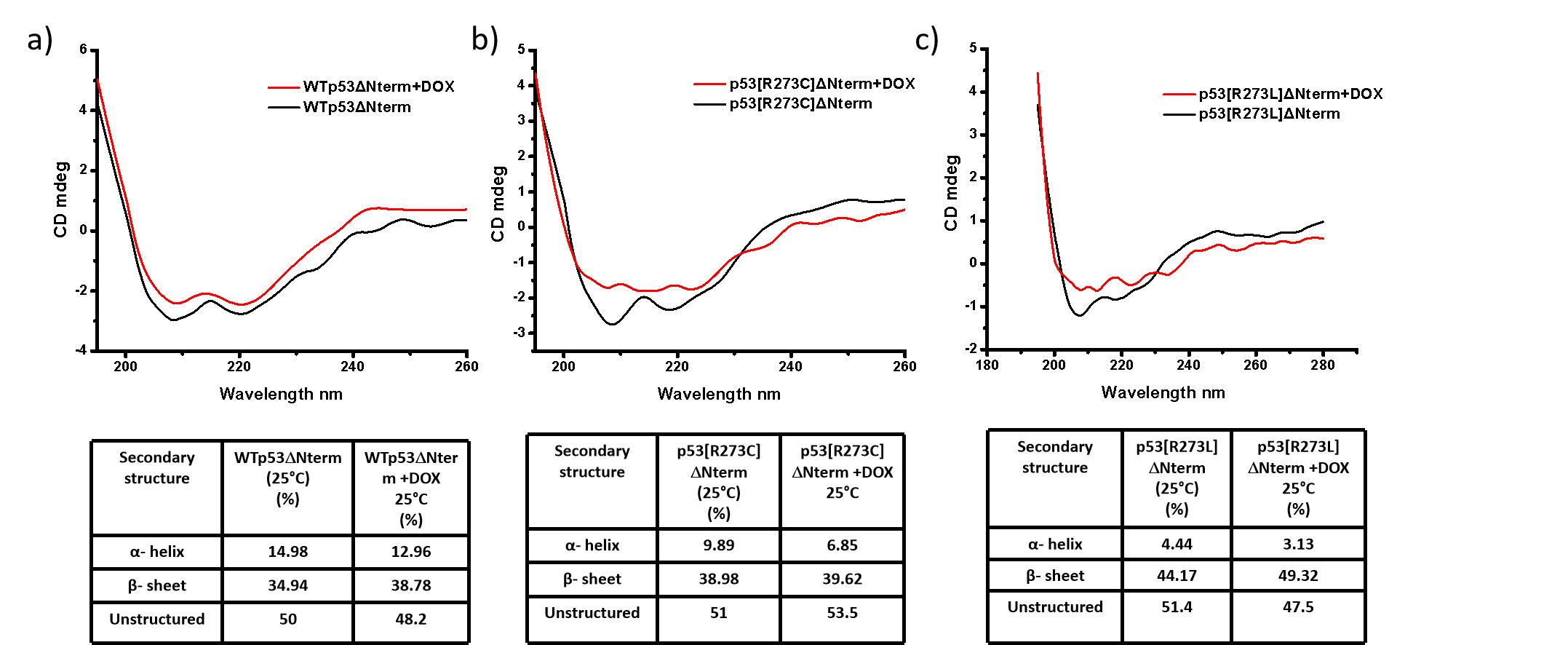


**Figure S2**: CD spectra of WTp53ΔNterm (a) and its R273 variants [R273C]p53 ΔNterm (b) and [R273L]p53 ΔNterm (c) in the presence (Red) and absence of doxorubicin (Black) at 25°C temperature respectively with a protein concentration of 5µM and doxorubicin concentration of 15µM at 150mM NaCl. K2D2 analysis showed distribution of secondary structure in WTp53 and its mutant variants in the presence and absence of doxorubicin.


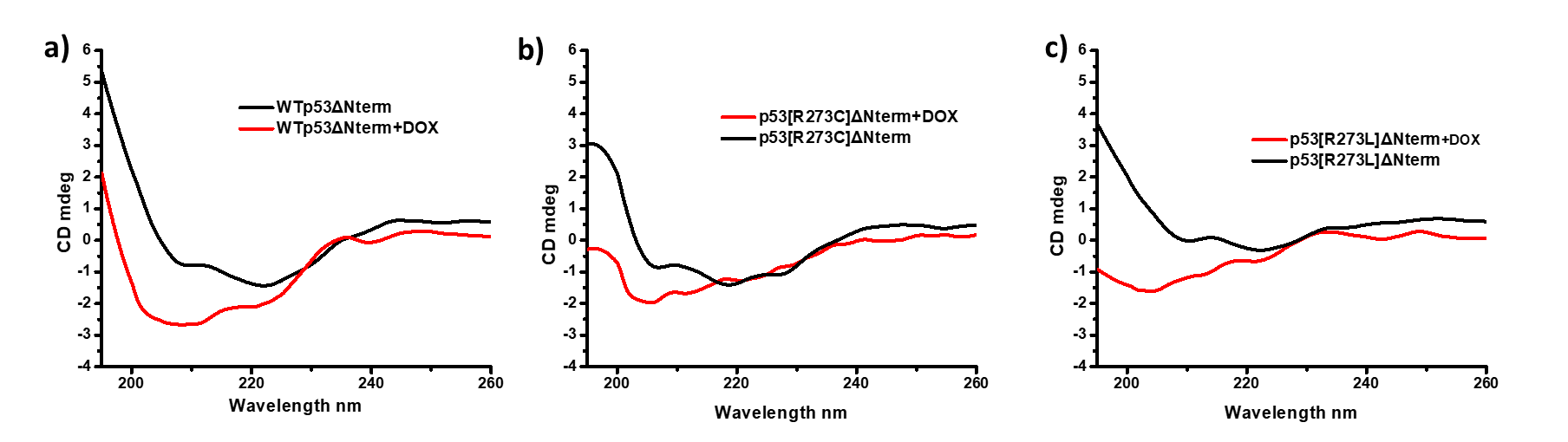


**Figure S3**: CD spectra of WTp53ΔNterm (a) and its R273 variants [R273C]p53 ΔNterm (b) and [R273L]p53 ΔNterm (c) in the presence (Red) and absence of doxorubicin (Black) at 37°C temperature respectively with a protein concentration of 5µM and doxorubicin concentration of 15µM at 150mM NaCl.

**
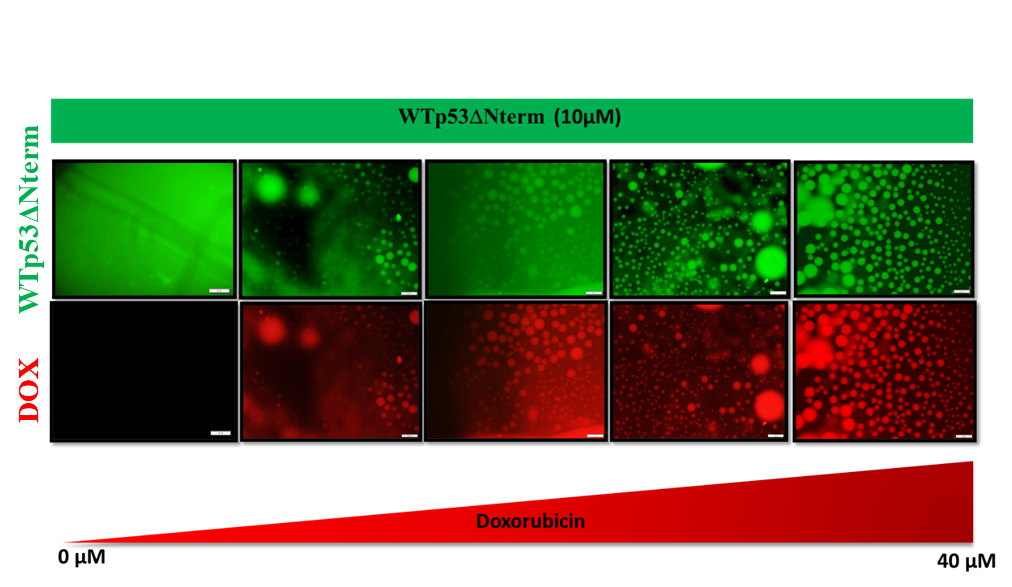
**

**Figure S4:** Co-phase separation of WTp53ΔNterm and doxorubicin with increasing concentration of doxorubicin in the presence of 5% of PEG-4000. Here, we have used protein concentration of 10µM, and doxorubicin range from 10µM to 40µM. The WTp53ΔNterm is labelled with Alexa-488 dye.

**Figure S5:** Turbidity assay of doxorubicin at different concentrations in PBS @pH7.4 @ 37°C temperature


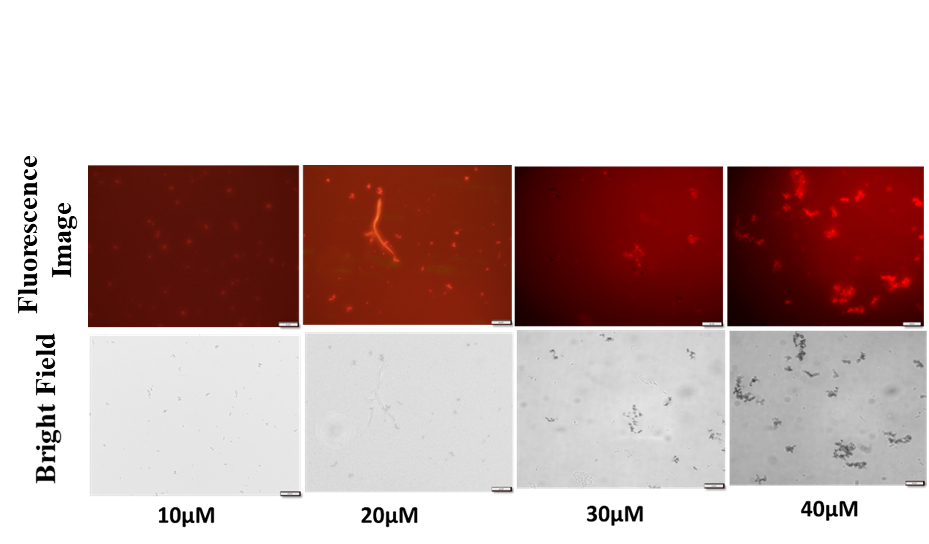


**Figure S6:** Microscopic images of doxorubicin in the presence of PEG-4000. No droplets are observed instead we observe some assemblies.


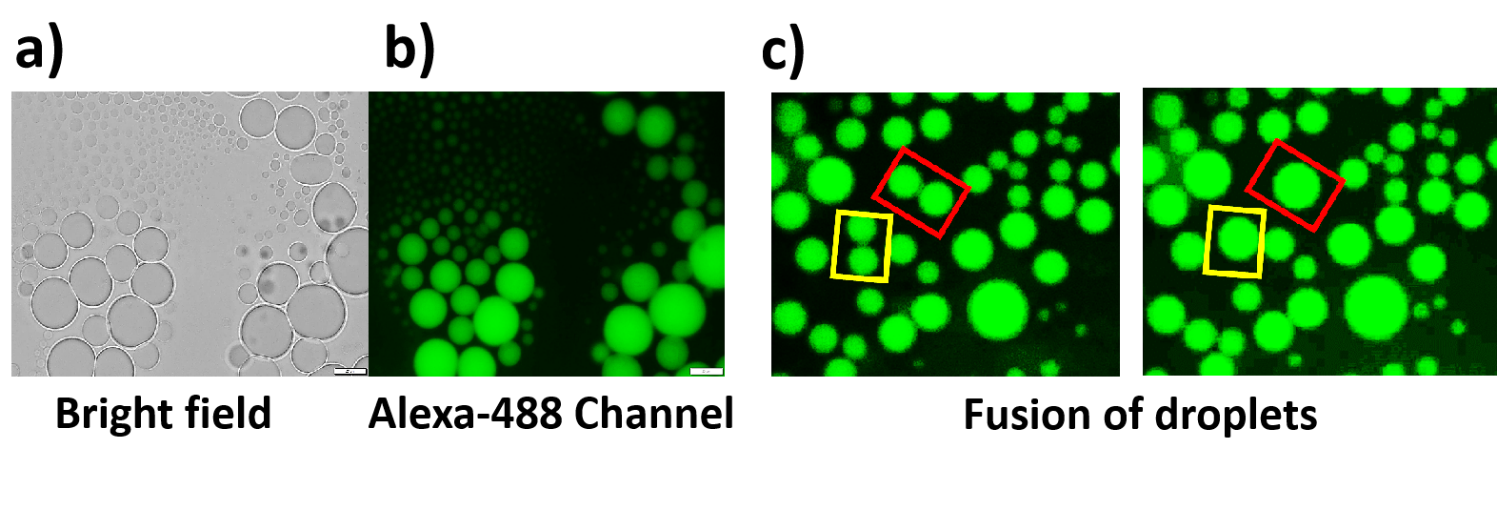


**Figure S7:** Microscopic images of Alexa-488 labelled [R273C]p53ΔNterm in the presence of 5% PEG-4000 at room temperature i.e., 25 °C. a) represents Bright field image, b) represents Fluorescent image in alexa-488 green. channel and c) shows fusion of the droplets representing liquid nature of the [R273C]p53ΔNterm condensates.


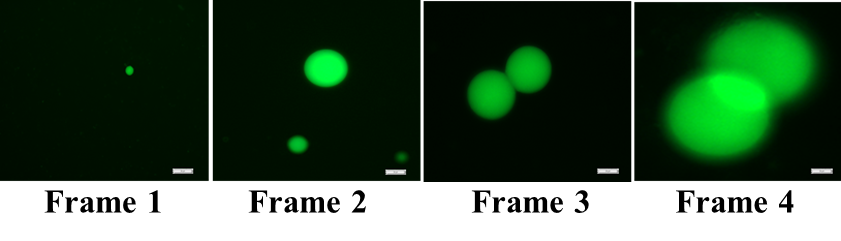

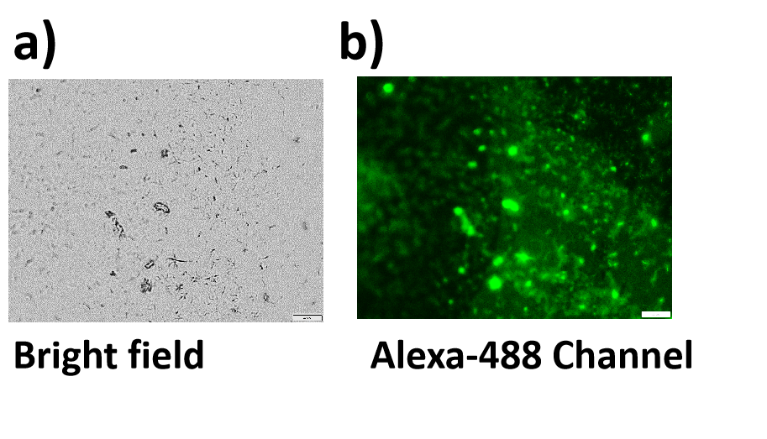


**c)**

**Figure S8**: The microscopic images of [R273L]p53ΔNterm @ 25 °C representing solid assemblies as shown as bright field image in a) and fluorescent image in b). [R273L]p53ΔNterm @ 18 °C formed liquid droplets as shown by droplets in c). Here, Different images are independent captured events of [R273L]p53ΔNterm in the presence of 5% of PEG-4000.

**
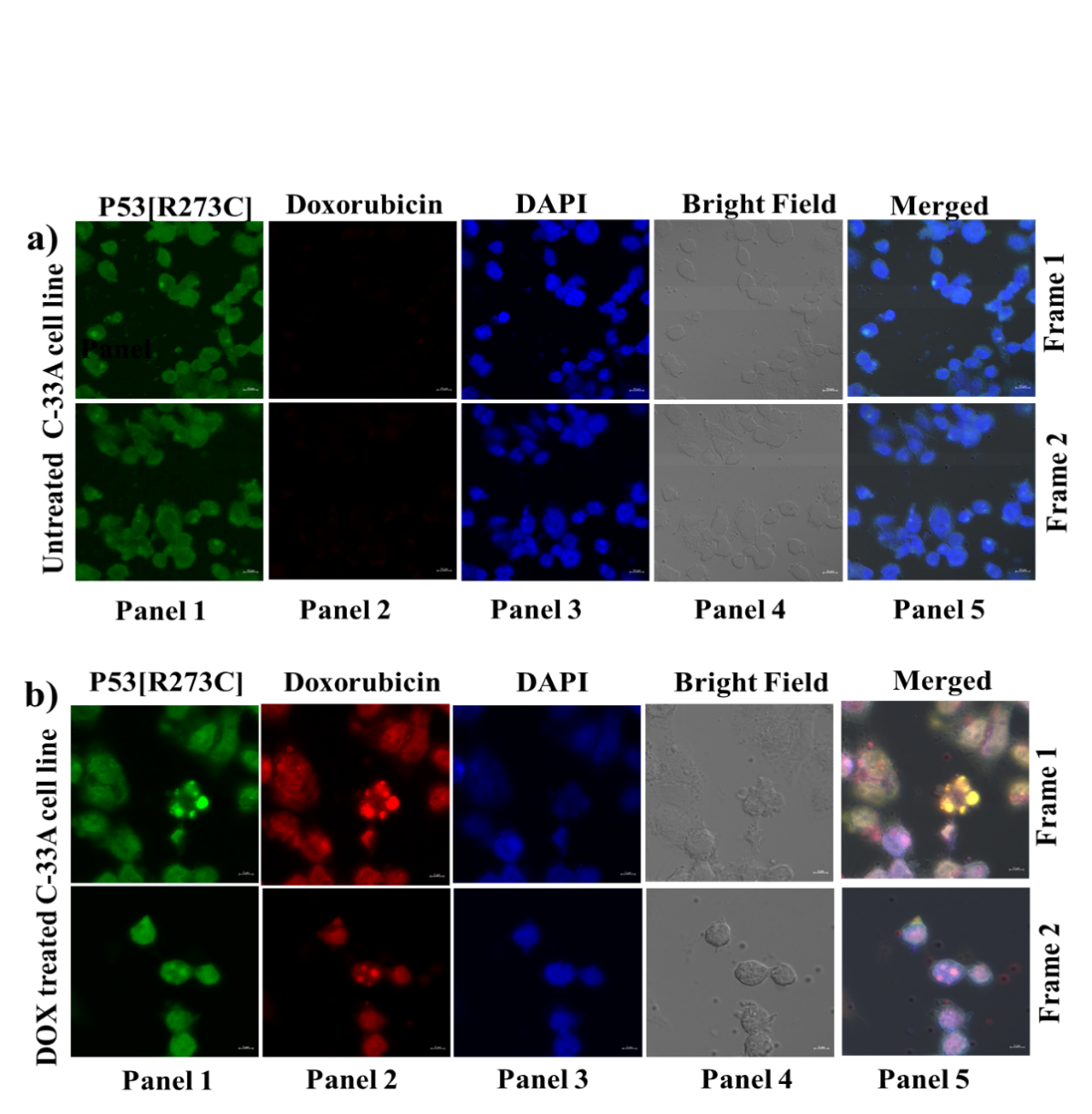
**

**Figure S9:** [R273C]p53 forms puncta in the untreated C-33A Cervical cell line (a); Colocalization of [R273C]p53 puncta and doxorubicin in the nucleus of the C-33A Cervical cell line in the doxorubicin treated cells (b) where Panel 1 is [R273C]p53; Panel 2 is Doxorubicin; Panel3 is DAPI; Panel 4 is bright field, and Panel 5 is merged image in a & b. We have taken two frames for each figure a & b.

**Explanation:** Cellular co-localization of p53 and doxorubicin: The effect of the doxorubicin on the p53 expression level and localization is observed using intrinsic fluorescence of doxorubicin and labelled antibody against endogenous p53. *In vitro* cellular studies show that in cancer cells, in the absence of doxorubicin treatment, p53 levels are very low and mainly localized in the cytoplasm of the cells. However, the treatment of doxorubicin increased the levels of p53. In addition to this, p53 colocalizes with the doxorubicin inside the nucleus in the treated cells. These observations are in support to the previous reports showing increase in the level of p53 upon doxorubicin administration (McSweeney et al., 2019). However, in case of [R273C]p53 containing untreated cell line C-33A, we observe puncta of [R273C]p53 indicating overexpression of oncogenic mutants in the cancerous cell line. In the presence of doxorubicin treatment, these puncta show co-localization with the doxorubicin in the nucleus of the cancer cells.

**Supporting Table**

| **Table S1: Residual interaction between p53 and doxorubicin obtained from Ligplot** | |
| --- | --- |
| **Protein –DOX** | **Interacting residues (type of interaction)** |
| **WTp53ΔNterm -DOX** | **Gly-199 ;(HYDB) Ser-227(HYDB); Tyr-233 (HYDB);**  **Asn-200(HB); Glu-221(HB); Glu-224(HB);**  **Thr-230(HB) and Thr-231(HB)** |
| **[R273C]p53ΔNterm -DOX** | **Gln-104(HYDB) ; Arg-110(HYDB) ; Leu-111(HYDB) ;**  **Phe -113(HYDB) ; Leu-114(HYDB) ; Tyr-126(HYDB) ;**  **Pro-128(HYDB) ; ASN-131(HYDB) ; Tyr-146(HYDB) ;**  **ASP-268(HYDB) ; SER-269 (HYDB)** |
| **p53[R273L]ΔNterm -DOX** | **Pro-98(HYDB) ; Ser-99(HYDB) ; Met-160(HYDB) ; Asp-208(HYDB) ; Asn-210(HYDB) ; Arg-158(HYDB) ; Ile-254(HYDB) ; Thr-256(HYDB) ; Glu-258(HYDB) ;**  **Val-97(HB) and Gly-262(HB)** |

**Note:** HB-hydrogen bonding and HYDB- hydrophobic interaction. Hydrogen bonding is shown in blue colour whereas hydrophobic contacts are shown in pink colour.

**Reference:**

McSweeney, K.M., Bozza, W.P., Alterovitz, W.-L., Zhang, B., 2019. Transcriptomic profiling reveals p53 as a key regulator of doxorubicin-induced cardiotoxicity. Cell Death Discov. 5, 102. https://doi.org/10.1038/s41420-019-0182-6
